# Brazilian Protocol for Sexually Transmitted Infections 2020: sexual violence

**DOI:** 10.1590/0037-8682-600-2020

**Published:** 2021-05-17

**Authors:** Maria Luiza Bezerra Menezes, Maria Alix Leite Araújo, Adriano Santiago Dias dos Santos, Elucir Gir, Ximena Pamela Díaz Bermúdez

**Affiliations:** 1 Universidade de Pernambuco, Departamento Materno-Infantil, Recife, PE, Brasil.; 2 Universidade de Fortaleza - UNIFOR, Programa de Pós-Graduação em Saúde Coletiva, Fortaleza, CE, Brasil.; 3 Ministério da Saúde, Secretaria de Vigilância em Saúde, Brasília, DF, Brasil.; 4 Universidade de São Paulo, Escola de Enfermagem de Ribeirão Preto, Ribeirão Preto, SP, Brasil.; 5 Universidade de Brasília, Departamento de Saúde Coletiva, Brasília, DF, Brasil.

**Keywords:** Sexual offenses, Intimate partner violence, Sexually transmitted diseases, Clinical protocols

## Abstract

This article addresses sexual violence, as part of the Clinical Protocol and Therapeutic Guidelines for Comprehensive Care for People with Sexually Transmitted Infections, published by the Brazilian Ministry of Health. Guidance is provided in programmatic and operational management, focusing on the service network for people in situation of sexual violence, recommendations to health staff about pregnancy and viral and non-viral sexually transmitted infections prophylactic measures, in addition to surveillance action strategies. Sexual violence is an encompassing issue that includes wider areas than the health field. It involves conceptual and programmatic challenges for health staff, at the forefront of care for affected people and also to the implementation of prevention strategies addressed to the whole society.

## HIGHLIGHTS

Sexual violence is one of the principal forms of human rights violation, affecting the right to life, health, and bodily integrity

## FOREWORD

This article addresses sexual violence, as part of the Clinical Protocol and Therapeutic Guidelines for Comprehensive Care for People with Sexually Transmitted Infections (STI), in addition to the PDCT on Post-Exposure Prophylaxis for Risk of HIV infections, STI and Viral Hepatitis, and the PDCT on HIV Infections Management in Children and Adolescents, published by the Brazilian Ministry of Health. The PDCT for Comprehensive Care for People with STI was approved by the National Committee for the Incorporation of Technologies (CONITEC) in the Brazilian National Health System (SUS)^1^ and updated by the group of specialists in STI in 2020. 

## INTRODUCTION

Sexual violence is defined as "any sexual act, attempt to obtain a sexual act, unwanted sexual comments or advances, or acts to traffic, or otherwise directed, against a person's sexuality using coercion, by any person regardless of their relationship to the victim, in any setting, including but not limited to home and work"^2^. 

Sexual violence is an intrinsic issue in several contemporary societies, often neglected. It can affect both sexes and all age groups, including severe physical consequences and emotional trauma for both victims and their families^3^. It is a multidimensional phenomenon, present in every social class, race, ethnic group, gender relation, and sexual orientation. It is one of the principal forms of human rights violation, affecting the right to life, health, and physical integrity^4^ ^-^ ^5^.

Facing sexual violence requires the participation of health, education, work, law enforcement, justice, and human rights bodies. It needs intersectoral public policies and integrated actions by the State and society in general. The role of respecting diversity and gender identities is highlighted, assuring access to rights in all areas and privileging the health of those affected^6^ ^-^ ^7^. 

One of the most serious - and most common - types of sexual violence is rape, defined as “physically forced or otherwise coerced penetration - even if slight - of the vulva or anus, using a penis, other body parts or an object”². Rape crime is defined in the Brazilian Criminal Code as the “act of embarrassing someone, through violence or serious threat, to have carnal conjunction or to practice or allow another libidinous act to be practiced”^8^.

Sexual violence has achieved notoriety. However, it is necessary to propose research agendas that contribute to scientific evidence in this field^6^ ^,^ ^9^ ^,^ ^10^. Records indicate that female individuals are the most affected, representing more than 80% of sexual violence victims^6^. In the period from 2011 to 2018, 1,282,045 cases of violence against women were reported^9^. 

Although scientific evidences point out that sexual violence occurs primarily among women, it also happens against men. For instance, a study that analyzed data on rape from 2017 to 2018 found that children aged from five to nine years old, were the most affected age group in males, representing around a quarter of the cases and the victimization peak occurred within boys aged seven years old^6^. Not only should the report of these events and their physical and psychosocial consequences be improved, but also it is suggested to develop studies to fill this empirical vacuum^11^. It can be observed that, although the constitution of masculinities predispose men not to recognize this type of violence, in the context of armed conflict, displacement, and migration sexual violence affecting men can occur in a higher incidence^12^ ^-^ ^14^.

Available data show that only 7.5% of the occurrence of sexual violence in 2019 in Brazil were reported to police authorities^15^; regarding rape, more than 60% of them are committed against vulnerable people, that is, people under 14 years old, considered legally unable to consent sexual intercourse. The same category refers to people unable to resist, regardless of their age, as someone under the effect of drugs, sickness, or dissability (Act No. 12,015, of August 7, 2009). It also shows the predominance of male aggressors in more than 80% of the cases^15^.

It is necessary to consider that sexual violence also affects transgender people, a social group of high vulnerability to violence and to sexually transmitted infections. Systematic review, based on 74 studies with this population, developed in Brazil and other countries, revealed that stigma and prejudice are related to gender identity and sexual orientation, expressed in the high prevalence of physical and sexual violence events against this social group^16^.

One of the sexual violence consequences is the possibility of STI transmission^17^, which causes fear and anxiety in victims, especially when it is related to the human immunodeficiency virus (HIV). For this reason, immediate assistance should be offered to the victim through clinical and laboratory care, post-exposure prophylaxis (PEP) on risk for HIV infection, viral hepatitis and non-viral STI (gonorrhea, syphilis, chlamydia infection, trichomonas infections, and cancer), psychological and social care, unintended pregnancy prevention, in addition to adequate guidance on medical procedures and legal rights^18^. 

A cross-sectional study that estimated the occurrence of pregnancy and STI due to sexual violence in the state of Santa Catarina between the years 2008 and 2013 identified that, 7,6% became pregnant, and 3,5% were affected by some STI^17^. The risk for such infections depends on the type of exposure (vaginal, anal, or oral), number of aggressors, exposure recurrence, presence of genital trauma, the victim's age, and their susceptibility (hymenal condition and previous presence of other STI)^17^.

Sexual violence, when committed by intimate partner, entails affective feelings which makes it more challenging to deal with. This is related to social, cultural, and economic context, such as naturalized values, ideologies, and norms^3^ ^,^ ^19^. Structured relationships, gender inequities, individual conditions and the social representation of violence give meaning to this phenomenon^20^. 

For such reasons, in addition to full knowledge on and implementation of therapeutic guidelines by health professionals, sexual violence victims must have assured access to the different support services for trauma recovery and healing process, with a comprehensive approach including physical, mental, and sexual health^21^.

## SEXUALLY TRANSMITTED INFECTION PROPHYLAXIS IN SEXUAL VIOLENCE SITUATIONS

Care within sexual violence events is urgent and access and support must be assured, recognizing key and priority populations' specificities. Such service must be offered in an appropriate place, with privacy assurance and no moral judgments. An initial assessment of the patient must include a comprehensive approach of the violent event and the pertinence of a prophylaxis prescription^22^ ^,^ ^23^.

**FIGURE 1:**
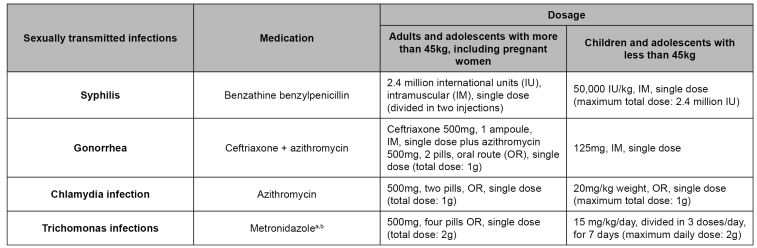
Prophylactic regimen for non-viral sexually transmitted infections in sexual violence situations.

Source: adapted from the Clinical Protocol and Therapeutic Guidelines for Comprehensive Care for People with Sexually Transmitted Infections^20^.

**Notes:** a) Prophylactic administration of metronidazole or its alternatives can be postponed or avoided in cases of known gastrointestinal intolerance to the drug.It must be postponed in cases when there is an emergency contraception prescription and post-exposure prophylaxis; b) Metronidazole cannot be used in the first trimester of pregnancy.

Immediate beginning of non-viral STI prophylaxis is recommended in all sexual violence cases, whenever possible^22^ and among pregnant women prophylactic administration is recommended at any gestational age^18^. The prophylactic regimen can be postponed depending on conditions that hinder adherence, such as people under extreme physical or emotional fragility. It can be avoided in cases of gastrointestinal intolerance to medications. Non-viral STI prophylactic regime in sexual violence situations is shown in Figure 1.

**FIGURE 2:**
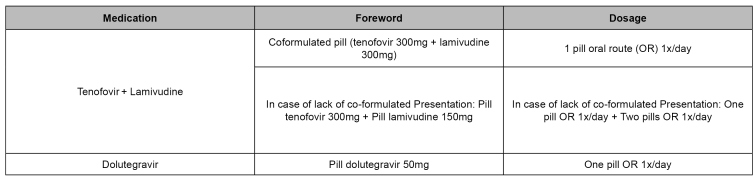
Preferential antiretroviral presentation and dosage for post-exposure prophylaxis.

**Source:** adapted from the Clinical Protocol and Treatment Guidelines for Post-Exposure Prophylaxis of Risk of HIV, STI and Viral Hepatitis Infections^15^.

**Note:** a) Contraindication for people with acute renal insufficiency and during preconception care; or women of childbearing age, discard pregnancy and indicate regularly using efficient contraceptive; not recommended for people using phenytoin, phenobarbital, oxcarbazepine, carbamazepine, dofetilide, and pilsicainide.

Post-exposure prophylaxis (PEP) at risk for HIV infection (PEP), viral hepatitis, and other STIs is a prevention strategy offered by the Brazilian National Health System (SUS). It consists of adopting drugs to reduce the risk of acquiring such infections^18^ ^,^ ^24^. PEP for HIV in Brazil is provided as one of the combine prevention strategies available for expanding intervention to prevent new HIV infections^18^. Combined prevention unites conjugates different actions and includes the combination of biomedical, behavioral, and structural interventions that can be applied in a individual or collective scope^25^. PEP for HIV prescribes antiretrovirals treatment during a period of 28 days and should be started no later than 72 hours after exposure. The preferred schema must include combinations of three or four antiretrovirals. It must be composed of two nucleoside reverse transcriptase inhibitors, preferably co-formulated, associated with another class, usually integrase inhibitors, preferably dolutegravir, or protease inhibitors with ritonavir, as a pharmaceutic adjuvant^18^ ^,^ ^26^ ^,^ ^27^. Presentation and dosages of preferred antiretrovirals recommended in Brazil for PEP, are shown in Figure 2 ^18^.

For pregnant women the preferred option for antiretroviral therapy regimen must be tenofovir and lamivudine, jointed with atazanavir/ritonavir in doses of atazanavir 300mg (one tablet) and ritonavir 100mg (one tablet), oral route, both once a day, or raltegravir in 400mg dose from the 14th gestational week (one tablet, oral route, twice a day)^18^.

Breastfeeding women must be informed about the potential risks of transmission of HIV through breast milk^28^ ^,^ ^29^. In the context of sexual violence, the temporary interruption of breastfeeding should be advised. During the immunological window period, extracting and discard milk is recommended; once an HIV control test has been carried out in the 12th week after starting PEP and its result is non-reactive, breastfeeding reintroduction is authorized^18^.

In clinical practice, sexual abuse diagnosis in babies, children, and adolescents is complex, and it also depends on the health professional's awareness and sensitivity^30^. Behaviors that may indicate sexually abused children include sexual perpetration age-inappropriate way of playing, (such as repeatedly touching an adult's genitals or asking an adult to touch their genitals)^30^. 

Despite such manifestations, evidence indicates that children's sexual violence remains invisible to health staff^31^. Children are more vulnerable to STI due to the anatomical and physiological immaturity of their genital-anal mucosa, among other causes. The diagnosis of an STI in children can be the first sign of sexual abuse and must be investigated^32^ ^,^ ^33^. Most complaints are nonspecific; however, rectal or genital trauma or bleeding and STI not acquired in the perinatal period due to vertical transmission must draw the health professional's attention^33^.

Regimens and dosages of PEP for HIV for this age group must be adjusted, especially in children under 12^15^. For children over 12, considering prescription safety and ease, tenofovir and lamivudine associated with dolutegravir are recommended^18^ ^,^ ^34^.

It should be noted that adolescents are entitled to PEP even without their parents or legal guardians' presence. In such cases, as provided for in the Child and Adolescent Statute - Act No. 8,069, of July 13, 1990 - the adolescent's discernment must be assessed, except in violent situations^35^.

If the person in a violent situation reports that they have not been vaccinated or have an incomplete vaccination schedule for hepatitis B, the first dose of the vaccine must be administered, or the vaccination schedule completed. Routine use of human anti-hepatitis B immunoglobulin (IGHAHB) is not recommended, unless the victim is susceptible and the person responsible for the violence is reactive to the hepatitis B virus surface antigen (HBsAg) or belongs to a risk group, such as, for example, people who use illicit drugs. When indicated, IGHAHB should be applied as early as possible, up to a maximum of 14 days after exposure^18^.

## PREGNANT PREVENTION IN SEXUAL VIOLENCE CONTEXTS

Guidelines on preventing unintended pregnancy in women´s victims under sexual violence care are available. For those deciding on this prophylaxis, levonorgestrel must be prescribed and offered in one 1.5mg tablet, oral route, or two 0.75mg tablets, single dose (or divided into two doses every 12 hours), up to five days after intercourse. This method has advantages in comparison with the Yuzpe method (administration of combined hormonal contraceptives as 200mcg of ethinylestradiol and 1mg of levonorgestrel in a single dose or divided into two doses, with an interval of 12 hours)^36^, given its greater effectiveness, since it fails in only 2% to 3% of cases, and safety, due to the lower occurrence of drug interactions and side effects^36^ ^,^ ^37^. 

When sexual violence inevitably leads to pregnancy, abortion is permitted by Decree-Law No. 2,848, of December 7, 1940, article 128, item II of the Brazilian Criminal Code, and other non-legal rules^8^ ^,^ ^38^. 

## SERVICE NETWORK ASSISTANCE AND ARTICULATION IN SEXUAL VIOLENCE SITUATIONS

Assistance to people under sexual violence situations in (SUS) must be offered following the current technical standard by the Ministry of Health^21^. It must be carried out as per Act No. 12,845, of August 1, 2013^39^ (provides for mandatory and comprehensive care by SUS), Decree No. 7,958, of March 13, 2013^40^ (sets forth guidelines for assistance by Law Enforcement professionals and SUS), and art. 5 of Ordinance GM/MS No. 485, of April 1, 2014^41^ (redefines the functioning of the service of assistance to people in such situation). 

Health service, in general, can represent a privileged space for the identification of a people in situations of sexual violence, provided that the health professionals are sensitive and attentive to specific signs and symptoms presented during consultations. Once sexual that offers emergency, comprehensive and multidisciplinary care. In this type of consultation, the patient must be is welcomed by a multidisciplinary team for receiving medication and advice. 

We highlight the role of listening and recording the violence event including clinical, gynecological, laboratory tests, evidence collection, emergency contraception, prevention for viral (HIV and hepatitis B) and non-viral STI, compulsory epidemiological notification in 24 hours (violence notification form), social, psychological and outpatient follow-up^22^ ^,^ ^42^.

Appropriate and quality care for victims of sexual violence demands structuring care networks with multidisciplinary teams, support at local level and implementation of protocols. Government agencies responsible for health and safety policies must identify the organizations and services available in the territory for this type assistance, such as: Women's Police Station, Child and Adolescent Police Station, Child Protection Council, Child, and Adolescent Rights Council, Reference Center for Social Assistance, Forensic Institute, Prosecution Office, shelters, women's groups, kindergartens, among others. Flow and access and case management problems at each level of this network need to be discussed and planned periodically^43^ ^,^ ^44^.

Even if the local level for comprehensive care of people affected by sexual violence, it is must be connected to neighboring cities for ensuring follow-up in specialized services^7^.

**FIGURE 3:**
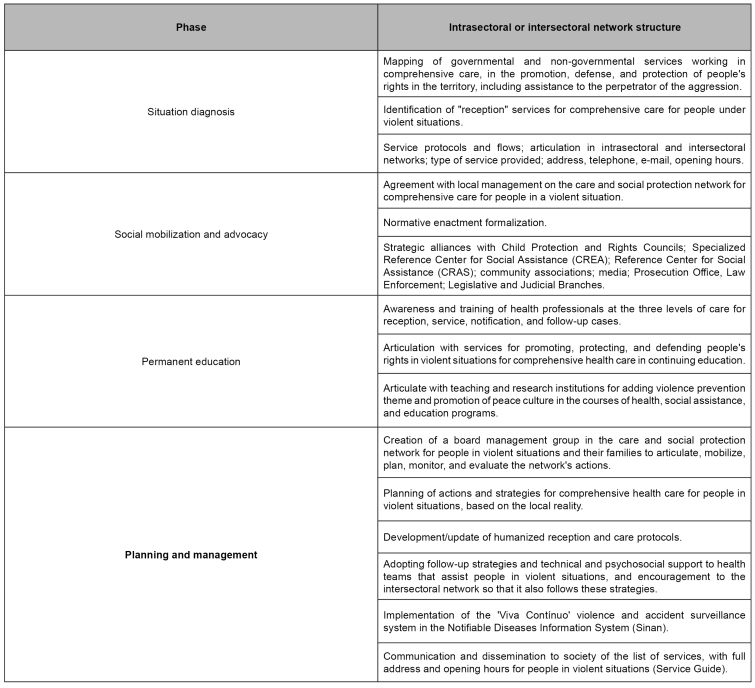
Structure of the intrasectoral and intersectoral care and social protection network for people in sexual violence situation.

**Source:** Clinical Protocol and Therapeutic Guidelines for Comprehensive Care for People with Sexually Transmitted Infections^20^.

Figure 3 shows some essential steps for materializing care and social protection network (intersectoral or intersectoral), which do not necessarily follow a hierarchy and can happen concurrently. Services for the care of people under sexual violence situations must be registered in the National Registry of Health Establishments System (SCNES), under code 165^44^. 

Health institutions must inform the availability of: (i) comprehensive care for people under sexual violence situations (classification 001 of specialized service 165), which can be organized in general hospitals and maternity hospitals, emergency services, Emergency Care Centers (UPA), and in the set of non-hospital emergency services, which must work 24 hours a day, and have a multidisciplinary team; (ii) outpatient care for people under sexual violence situation (classification 007 of specialized service 165); and (iii) pregnancy termination in the cases provided for by law (classification 006 of technical assistance 165)^44^.

Regarding assistance to people in sexual violence situation, compulsory notification stands out, as determined by Ordinance GM/MS No. 1,271, of June 6, 2014^45^, and Ordinance GM/MS No. 204, of February 17, 2016 ^46^, and the provisions of Act No. 8,069, of July 13, 1990 (provides for the Child and Adolescent Statute and other requirements)^35^, in Act No. 10,778, of November 24, 2003 (establishes compulsory notification, in national territory, of the case of violence against women treated in public or private health services)^47^, and Act No. 10,741, of October 1, 2003 (provides for the Elderly Statute and sets other measures)^48^.

In case of sexual violence and suicide attempts, the notification must occur within 24 hours at the local level, aiming to ensure timely intervention in such cases^45^ ^,^ ^46^. Immediate record is essential for service organizations and provides timely access to the disease's prevention measures above. The notification shall take place from the flow defined by local surveillance: the health service fills out the specific form of the Notifiable Diseases Information System (Sinan) and forwards it to municipal surveillance, which follows the flow to the state surveillance and, later, to the Health Surveillance Department of the Ministry of Health^45^ ^,^ ^46^.
